# Supplementary material for: Scavenger: A pipeline for recovery of unaligned reads utilising similarity with aligned reads
Source: F1000Res. 2022 Oct 13;8:1587. Originally published 2019 Sep 4. [Version 2] doi: 10.12688/f1000research.19426.2 (PMC7459848; doi:10.12688/f1000research.19426.2)
Supplement: Peer review history

# Peer review: Reviewer response for version 1

**DOI:** 10.5256/f1000research.21296.r68338

**Published:** 2020-08-27

**Authors:** Marco Previtali, Luca Denti

**Affiliations:** 1 Department of Computer Sciences, Systems and Communications, University of Milan-Bicocca, Milan, Italy

Yang *et al.* present Scavenger, a tool for rescuing unaligned RNA-Seq reads by exploiting information from reads which are successfully aligned. Scavenger is split into four main steps and can be easily run on cloud-based computing platforms.

 We think that the problem tackled in this paper is interesting and that the tool proposed may improve the overall accuracy of any downstream RNA-Seq analysis. Indeed, many studies have shown that unaligned reads carry important information that is usually discarded from RNA-Seq analysis pipelines, thus recovering such reads will improve their results and shed more light on the dataset under analysis.

 Overall the paper is easy to read and the authors clearly state the problem they are tackling.

 The method is well described and a big part of the paper is devoted to an extensive evaluation of the tool using real data.

**Major remarks:**

 We have a single main remark for the authors.

 Although we appreciate the extensive experimental evaluation, it is not easily reproducible.  We suggest the authors publish the commands used to run all the experiments in a supplementary repository or document, ideally in the form of a Snakemake or Nextflow pipeline.

 Moreover, in Section “Dataset” the authors report the commands used to run STAR, subread-align, and blastn.  We think that it is not useful to report them in the manuscript and we suggest to remove them for better readability.

**Minor remarks and typos:**

- Page 4: "(i.e. the number of aligned reads that fall within the interval)" -> which aligned reads? the unaligned realigned or the aligned?
- Page 4: please describe (intuitively) what “realignability of mapped reads” and “non-realignability of unmapped reads” means.
-  Page 6: "mousub" > “mouse”?
- Page 6-7: command lines are ugly (maybe due to two columns page format).
- Section “Results”: it would be useful to describe the running times and the RAM requirements of Scavenger in the various experimental analysis to better understand whether it can be run on low-end and mid-range servers.
- Table 6 reports the results of different experiments described in the manuscript. The last sentence on page 9 might be confusing since it states that the minimum number of rescued reads is 17,000 whereas the table includes two smaller values (12,347 and 5,368).  We suppose that these two lines refer to the results on Mouse scRNA reads. We suggest the authors to better specify the experiment on each line (or split the table in two).
- Table 6 reports the results for all the samples considered in the evaluation (human and mouse). We believe that the authors should specify the organism for each accession ID.
- Page 10: We concur with the other reviewer and we think that the sample size for random sampling of reads is too small relative to the total mapped reads.  The authors should improve this part of the manuscript.
- Page 10: "11 samples originating from two human projects" -> there is no table. I would like to see it (as done for the mouse samples).
- Figure 3: the caption is wrong. There is no B subfigure. Moreover, the axes have no labels making the charts harder to understand.
- Page 12: “and the typically low expression of pseudo- genes which is therefore more affected by increase an in reads as a result of recovery by Scavenger” -> please rephrase this sentence since it’s not completely clear to us.
- Page 15: "are able produce" -> "are able to produce"

Are the conclusions about the tool and its performance adequately supported by the findings presented in the article?

Yes

Is the rationale for developing the new software tool clearly explained?

Yes

Is the description of the software tool technically sound?

Yes

Are sufficient details of the code, methods and analysis (if applicable) provided to allow replication of the software development and its use by others?

Partly

Is sufficient information provided to allow interpretation of the expected output datasets and any results generated using the tool?

Partly

Reviewer Expertise:

algorthmic bioinformatics

We confirm that we have read this submission and believe that we have an appropriate level of expertise to confirm that it is of an acceptable scientific standard.

# Response

**Published:** 2022-07-31

**Authors:** Andrian Yang

**Affiliations:** EMBL European Bioinformatics Institute, UK

We thank the reviewer for their overall positive assessment of our work, as well as their constructive comments. Please find below our point-by-point response to the reviewer's comments:

*Major remarks:*

*We have a single main remark for the authors.*

*Although we appreciate the extensive experimental evaluation, it is not easily reproducible.  We suggest the authors publish the commands used to run all the experiments in a supplementary repository or document, ideally in the form of a Snakemake or Nextflow pipeline.*

 We thank the reviewer for the suggestion and will be happy to share commands used with viewers upon request. We will endeavour to make all code available upon publication in future studies.

*Moreover, in Section “Dataset” the authors report the commands used to run STAR, subread-align, and blastn.  We think that it is not useful to report them in the manuscript and we suggest to remove them for better readability.*

 While we thank the reviewer for the suggestion, we believe the inclusion of the commands used are helpful for the viewer to understand the alignment parameter used in each of the program as the result of the alignment may differ depending on the alignment parameters used.

*Minor remarks and typos: *

- *Page 4: "(i.e. the number of aligned reads that fall within the interval)" -> which aligned reads? the unaligned realigned or the aligned?*

*We have added a clarification in the section highlighted by the reviewer. *

- *Page 4: please describe (intuitively) what “realignability of mapped reads” and “non-realignability of unmapped reads” means.*

The requirement of realignability of mapped reads is for reads which are mapped in one run of the alignment to also be mapped in other runs of the alignment if no other alignment parameters are modified. Similarly the definition of non-realignability of unmapped reads is for reads which are unmapped in one run of the alignment to also still be unmapped other runs of the alignment if no other alignment parameters are modified. These requirements differs from the deterministic alignment property, as some (multi) mapped reads may be assigned to different mapping location in different runs of the alignment even with identical alignment parameters.

- * Page 6: "mousub" > “mouse”?*

The mousub term used here refers to the name of the sequence file provided by Repbase for mouse organism. We have added extra clarification in the manuscript.

- *Page 6-7: command lines are ugly (maybe due to two columns page format). *
- *Section “Results”: it would be useful to describe the running times and the RAM requirements of Scavenger in the various experimental analysis to better understand whether it can be run on low-end and mid-range servers.*

We unfortunately did not record the running times for scavenger across the experimental analysis so we are not able to include this information within the manuscript. The RAM requirement of Scavenger is largely dictated by the RAM requirement for the alignment tool used (~30GB for human genome to run STAR), though it may also scale depending on the number of reads processed.

- *Table 6 reports the results of different experiments described in the manuscript. The last sentence on page 9 might be confusing since it states that the minimum number of rescued reads is 17,000 whereas the table includes two smaller values (12,347 and 5,368).  We suppose that these two lines refer to the results on Mouse scRNA reads. We suggest the authors to better specify the experiment on each line (or split the table in two).*

The amount of reads recovered as alluded to in the last sentence on page 9 refers to the first section of the table, which give statistics for the datasets from selected non-reference mouse strain. We have added extra clarification in the figure caption.

- *Table 6 reports the results for all the samples considered in the evaluation (human and mouse). We believe that the authors should specify the organism for each accession ID.*

We have added extra clarification in the figure caption.

- *Page 10: We concur with the other reviewer and we think that the sample size for random sampling of reads is too small relative to the total mapped reads.  The authors should improve this part of the manuscript.*

We thank the reviewer for their feedback regarding the experiment where we evaluated the degree of polymorphism between source aligned and scavenger recovered reads. We will endeavour to perform more rigorous experiment as suggested by the reviewer in future studies.

- *Page 10: "11 samples originating from two human projects" -> there is no table. I would like to see it (as done for the mouse samples).*

The details of the human samples used in this study is included in Table 1.

- *Figure 3: the caption is wrong. There is no B subfigure. Moreover, the axes have no labels making the charts harder to understand.*
- *Page 12: “and the typically low expression of pseudo- genes which is therefore more affected by increase an in reads as a result of recovery by Scavenger” -> please rephrase this sentence since it’s not completely clear to us.*
- *Page 15: "are able produce" -> "are able to produce"*

We have now fixed the sections highlighted by the reviewers following the reviewers’ suggestions.

# Peer review: Reviewer response for version 1

**DOI:** 10.5256/f1000research.21296.r53438

**Published:** 2019-10-21

**Authors:** Quan Nguyen

**Affiliations:** 1 Institute for Molecular Bioscience, The University of Queensland, Brisbane, Qld, Australia

The authors present Scavenger, a pipeline to recover false unaligned reads by remapping or BLAST-search unmapped reads that are similar to aligned reads. The false unaligned reads commonly arise from two sources: the actual sequence variation to the shared reference genome and the mapping to multiple regions. These reads are usually discarded in a standard analysis pipeline. 

 The software can be useful to rescue unmapped reads in a quantifiable way, which can be important in many sequencing data analysis scenarios. Example usage cases are to study the expression of frequently mutated genes in diseased samples and in the mapping cases where a genome reference is not complete or high divergence to the reference exists.

 The authors comprehensively tested Scavenger using three simulation settings, 80 RNA-seq datasets from non-reference inbred laboratory mouse strains, 11 human and 12 mouse datasets and 80 single-cell datasets. They performed analyses on the effects of recovering falsely unaligned reads to downstream analyses and show that pseudogenes affect expression measurements. Scanvenger input files are FASTQ files, or mapped BAM/SAM files, and the software has functionalities for parallelisation on CPUs and AWS cloud processing. 

 I suggest some minor revisions below: 

- FASTQ read statistics like length and quality should be described. What preprocessing steps are required, for example trimming of N bases, filtering of low-quality reads? While multiple mapping and divergence in genome sequence are two primary sources of false unaligned reads, another common source can be from sequencing base calling error. The authors may add discussion on how quality scores affect read recovery. 
- The tests for difference in the level of polymorphism between unligned and aligned reads are interesting and can lead to important conclusions. These tests can be improved. The sample size from a random sampling of reads (1000 reads) is small relative to the total mapped reads (< 0.1%), and t-test for results from 10 iterations is not suitable to test for testing the enrichment of SNPs in aligned vs unaligned reads. The authors may consider the permutation test or others. The authors may also consider known regions of the genome where more mutations are expected. 
- The recovery of un-detected genes and effects on fold-change are interesting. The author may consider adding information or a panel in figure 5 to show expression levels of these affected genes before and after read recovery. 
- Page 4: clarify "qualities" as mapping or sequencing qualities. 
- Page 4: clarify "to reduce potential location for alignment". Can be useful to output total reads that can not be rescued due to multiple mapping. 
- Tables 2 and 3, the unaligned category on the third column should be a part of the source execution.
- Add discussion on when Scavenger should be used as an alternative for parameter optimisation approach in STAR alignner. For example, on Pages 8 and 15, the authors dicuss "performing parameter optimisation is not trivial due to lack of ground truth", how does Scavenger perform better in the absence of ground truth?  Would it be due to speed, accuracy and quantitativeness? 
- Figure 3, add label A) and B), and description for panel titles and graph axis titles.
- Page 12, what single-cell sequencing platforms were used for the selected datasets. Variation in recovery efficient may occur in, for example, 3'-sequencing and full-length sequencing data. 

Are the conclusions about the tool and its performance adequately supported by the findings presented in the article?

Yes

Is the rationale for developing the new software tool clearly explained?

Yes

Is the description of the software tool technically sound?

Yes

Are sufficient details of the code, methods and analysis (if applicable) provided to allow replication of the software development and its use by others?

Yes

Is sufficient information provided to allow interpretation of the expected output datasets and any results generated using the tool?

Partly

Reviewer Expertise:

Transcriptomics, genomics, machine-learning, single-cell

I confirm that I have read this submission and believe that I have an appropriate level of expertise to confirm that it is of an acceptable scientific standard.

# Response

**Published:** 2022-07-31

**Authors:** Andrian Yang

**Affiliations:** EMBL European Bioinformatics Institute, UK

We thank the reviewer for their overall positive assessment of our work, as well as their constructive comments. Please find below our point-by-point response to the reviewer's comments:

- *FASTQ read statistics like length and quality should be described. What preprocessing steps are required, for example trimming of N bases, filtering of low-quality reads? While multiple mapping and divergence in genome sequence are two primary sources of false unaligned reads, another common source can be from sequencing base calling error. The authors may add discussion on how quality scores affect read recovery. *

We would like to highlight Table 6 to the reviewer which contains the read length statistics for each sample. Unfortunately, the read quality information is not readily available so we are not able to provide the statistics in the manuscript. With regards to read pre-processing, we performed basic pre-processing of sequencing reads using Trimmomatic to remove low quality sequence and short reads, though recent studies have shown that read pre-processing may not be required for RNA-seq alignment as demonstrated in Liao and Shi 2020 ( https://academic.oup.com/nargab/article/2/3/lqaa068/5901066). We agree with the reviewer that low quality reads likely contribute to false unaligned reads by aligner due to increased mismatches/indel in read sequence as previously described by Dobin and Gingeras 2015 ( https://currentprotocols.onlinelibrary.wiley.com/doi/10.1002/0471250953.bi1114s51). However,  since the recovery process largely re-utilise the aligner used in the initial alignment (source execution), the recovery rate of low quality sequences are likely to be low as the recovery process imposes the same minimum sequence requirement for recovery.

  

- *The tests for difference in the level of polymorphism between unligned and aligned reads are interesting and can lead to important conclusions. These tests can be improved. The sample size from a random sampling of reads (1000 reads) is small relative to the total mapped reads (< 0.1%), and t-test for results from 10 iterations is not suitable to test for testing the enrichment of SNPs in aligned vs unaligned reads. The authors may consider the permutation test or others. The authors may also consider known regions of the genome where more mutations are expected. *

We thank the reviewer for their feedback regarding the experiment where we evaluated the degree of polymorphism between source aligned and scavenger recovered reads. We will endeavour to perform more rigorous experiments as suggested by the reviewer in future studies.

- *The recovery of un-detected genes and effects on fold-change are interesting. The author may consider adding information or a panel in figure 5 to show expression levels of these affected genes before and after read recovery. *

We would like to highlight Figure 4 to the reviewer, which provide a summary of the gene expression before and after read recovery for the three different type of datasets.  

- *Page 4: clarify "qualities" as mapping or sequencing qualities. *
- *Page 4: clarify "to reduce potential location for alignment". Can be useful to output total reads that can not be rescued due to multiple mapping. *
- *Tables 2 and 3, the unaligned category on the third column should be a part of the source execution.*

We have added clarifications to the sections that the reviewer highlighted and fixed the table layout.

- *Add discussion on when Scavenger should be used as an alternative for parameter optimisation approach in STAR alignner. For example, on Pages 8 and 15, the authors dicuss "performing parameter optimisation is not trivial due to lack of ground truth", how does Scavenger perform better in the absence of ground truth?  Would it be due to speed, accuracy and quantitativeness?*

A typical alignment optimisation approach, especially in the absence of ground truth, is to minimise the amount of unaligned reads by increasing the edit distance threshold during alignment at the risk of increasing the number of incorrectly aligned read. While it is possible to find an optimised parameter which maximise the number of aligned reads while minimising the number of incorrectly aligned reads through varying different parameter values, this is not entirely possible to do in the absence of ground truth and will likely take a lot of time and effort to test all possible permutation of alignment parameters. Scavenger has been shown to be able to recover reads with reasonably high degree of accuracy without the need for extensive parameter optimisation, with the reads recovered having very degree of overlap to reads recovered with parameter optimisation, and as such, would be a good method to utilise after minimal or no parameter optimisation. We have added extra discussion in the manuscript.

- *Figure 3, add label A) and B), and description for panel titles and graph axis titles.*

We have now revised the caption for Figure 3 and added graph axis titles to the figure. 

- *Page 12, what single-cell sequencing platforms were used for the selected datasets. Variation in recovery efficient may occur in, for example, 3'-sequencing and full-length sequencing data. *

The single-cell sequencing platform utilised for generating the mouse brain scRNA-seq dataset by Zeisel *et al. *is the Fluidigm C1 system with a custom protocol using 5’ capture and tagging of RNA modules as described in Islam *et al.* 2013 ( https://www.nature.com/articles/nmeth.2772).
